# Complete Lower Eyelid Retractor Release in Selected Patients with End-Stage Involutional Ectropion: A Preliminary Retrospective Case Series

**DOI:** 10.3390/jcm15145619

**Published:** 2026-07-17

**Authors:** Daliborka Miletić, Biljana Kuzmanović Elabjer, Iva Bulat, Dora Marinčić, Mladen Bušić

**Affiliations:** 1 Department of Ophthalmology, Sveti Duh University Hospital, 10000 Zagreb, Croatia; dmiletic@kbsd.hr (D.M.); martincevic.dora@gmail.com (D.M.); mbusic@kbsd.hr (M.B.); 2 Faculty of Dental Medicine and Health Osijek, University Josip Juraj Strossmayer of Osijek, 31000 Osijek, Croatia; busiciva@kbsd.hr; 3 Faculty of Medicine Osijek, University Josip Juraj Strossmayer of Osijek, 31000 Osijek, Croatia

**Keywords:** severe involutional ectropion, lower eyelid retractor, retractor release

## Abstract

**Background**: Severe involutional lower eyelid ectropion with complete eyelid eversion represents an advanced stage of eyelid malposition in which long-standing involutional changes may be accompanied by secondary structural remodeling of the posterior lamella. Although conventional surgical techniques are generally effective in mild to moderate cases, the management of end-stage involutional ectropion remains challenging. **Methods**: This retrospective single-center case series included nine patients treated for severe involutional lower eyelid ectropion with complete eyelid eversion between January 2024 and December 2025. Surgical treatment consisted of horizontal eyelid tightening, with adjunctive procedures performed according to intraoperative findings. Lower eyelid retractors were managed by reattachment, tightening, or complete release based on intraoperative clinical judgment when marked fibrosis, shortening, and persistent inferior traction were identified. Clinical outcomes were analyzed descriptively. **Results**: Complete lower eyelid retractor release was performed in three patients during primary surgery and two during revision surgery. The remaining patients underwent retractor tightening or reattachment. During a mean follow-up of 10.5 months, recurrent ectropion occurred in five of six patients treated with retractor tightening or reattachment. No recurrence was observed after complete retractor release, either as a primary or revision procedure. Revision surgery including complete retractor release restored a stable eyelid position in all reoperated patients during the available follow-up period. **Conclusions**: Complete lower eyelid retractor release was associated with favorable anatomical outcomes in this small retrospective case series of selected patients with intraoperative evidence of marked retractor fibrosis or shortening. These findings are descriptive and hypothesis-generating and should be interpreted within the limitations of the study.

## 1. Introduction

Lower eyelid ectropion is characterized by outward rotation of the eyelid margin, resulting in loss of normal eyelid–globe apposition and impaired protection of the ocular surface. In advanced cases, complete eversion of the lower eyelid leads to conjunctival exposure, chronic epiphora, punctal malposition, keratinization of the palpebral conjunctiva, ocular surface irritation, and substantial functional and cosmetic impairment, significantly affecting patients’ quality of life [1,2].

Involutional ectropion is the most common subtype and results from progressive age-related degeneration of the anatomical structures responsible for lower eyelid stability. Degenerative changes involving the medial and lateral canthal tendons, attenuation of the orbicularis oculi muscle, and weakening or disinsertion of the lower eyelid retractors contribute to progressive horizontal and vertical eyelid instability [3,4,5,6,7,8,9]. In advanced disease, these pathological processes frequently coexist, creating a complex biomechanical disturbance that involves multiple components of the lower eyelid and complicates surgical management.

Several surgical techniques have been described for the treatment of involutional lower eyelid ectropion [6,10,11,12,13,14,15,16,17]. Conventional procedures primarily address horizontal eyelid laxity through lateral tarsal strip (LTS) surgery, wedge resection, or other shortening procedures, often combined with correction of medial eyelid abnormalities. These techniques generally provide satisfactory results in patients with mild or moderate involutional ectropion [10,12,13,14]. However, their effectiveness may be limited in severe cases characterized by complete lower eyelid eversion, particularly when pathological changes of the lower eyelid retractors contribute to persistent vertical imbalance, maintaining the everted position [3,4,18,19]. This highlights the need for surgical strategies that address both horizontal laxity and vertical vector forces within the eyelid complex [1,16,17,18]. During surgical management of advanced ectropion, some patients are found intraoperatively to have marked fibrosis and shortening of the lower eyelid retractors, which produce persistent downward traction despite adequate horizontal eyelid tightening. In such cases, complete release of the retractors may reduce or eliminate this abnormal traction and facilitate restoration of physiological eyelid position. Although this concept is supported by intraoperative observations, its role in the management of severe involutional ectropion has received little attention in the literature.

The present study describes our retrospective experience with surgical management of severe involutional lower eyelid ectropion with complete eyelid eversion. The primary aim was to describe the surgical technique and clinical outcomes associated with complete lower eyelid retractor release in selected patients with intraoperative evidence of retractor fibrosis or shortening. Given the retrospective design and limited number of patients, the findings are intended to generate hypotheses and inform future prospective studies rather than establish comparative treatment efficacy.

## 2. Materials and Methods

This retrospective single-center case series included consecutive patients who underwent surgical treatment for severe involutional lower eyelid ectropion at the Department of Ophthalmology between January 2024 and December 2025. Medical records, operative reports, and available clinical photographs were reviewed.

Patients were eligible if they presented with severe involutional lower eyelid ectropion characterized by complete fixed eversion of the lower eyelid, complete loss of eyelid–globe apposition, severe lateral canthal tendon laxity clinically manifested by rounding of the lateral canthus, punctal eversion, and persistent exposure of the palpebral conjunctiva. In all patients, the eyelid remained permanently everted and could not maintain spontaneous apposition to the globe. Because all patients presented with end-stage disease characterized by permanent eyelid eversion and complete lateral canthal insufficiency, additional functional tests commonly used in mild or moderate involutional ectropion, such as the snap-back and distraction tests, were not considered clinically informative and therefore were not routinely performed.

Patients with cicatricial, paralytic, congenital, or mechanical ectropion were excluded. Patients with mild or moderate involutional ectropion were also excluded. Although intraoperative fibrosis and shortening of the lower eyelid retractors were observed in several patients, none demonstrated clinical features of primary cicatricial ectropion. Patients with a history of facial burns, periocular trauma, previous lower eyelid surgery causing cicatricial shortening, inflammatory skin disease, or other causes of anterior lamella scarring were excluded.

All surgical procedures were performed under local anesthesia by one of two experienced oculoplastic surgeons (BKE or DM). The operative technique consisted of a combined approach addressing both horizontal and vertical components of eyelid malposition.

Horizontal eyelid instability was corrected using a lateral tarsal strip (LTS) procedure. Following lateral canthotomy and inferior cantholysis, a lateral tarsal strip was fashioned, shortened according to the degree of horizontal laxity, and secured to the periosteum of the lateral orbital rim at the level of the physiological lateral canthus [11,12,13,14]. In selected revision cases with marked residual horizontal laxity, full-thickness wedge excision was additionally performed. In patients with significant punctal eversion, a medial conjunctival diamond excision was carried out to improve punctal inversion and restore contact with the tear lake [11,20].

Vertical correction was performed according to the principles of the Jones procedure [11,20]. Following a subciliary skin incision and elevation of a skin–muscle flap, careful dissection was performed to expose the lower eyelid retractors. In patients in whom the retractors appeared mobile without significant fibrosis or shortening, the retractors were reattached or tightened using interrupted absorbable and non-absorbable sutures.

When, in the surgeon’s clinical judgment, dense fibrosis, shortening, reduced mobility of the retractors, and persistent inferior traction prevented passive repositioning of the eyelid despite correction of horizontal laxity, the retractors were completely released from the inferior tarsal border. Fibrotic adhesions were carefully divided until abnormal inferior traction on the lower eyelid was eliminated. Adequate release was confirmed intraoperatively when the lower eyelid could be repositioned against the globe without residual downward traction before horizontal fixation was completed. The retractors were not reattached to the tarsal plate following release, and only skin was closed with interrupted absorbable sutures.

Anterior lamella reconstruction was performed only when clinically significant skin deficiency prevented tension-free eyelid repositioning. In these patients, reconstruction was achieved using either a full-thickness skin graft or a flap harvested from the ipsilateral upper eyelid. A temporary Frost suture was used in selected cases to support the eyelid in the early postoperative period.

Given the limited number of patients and the descriptive nature of the study, only descriptive statistical analyses were performed.

## 3. Results

**Figure 1:**
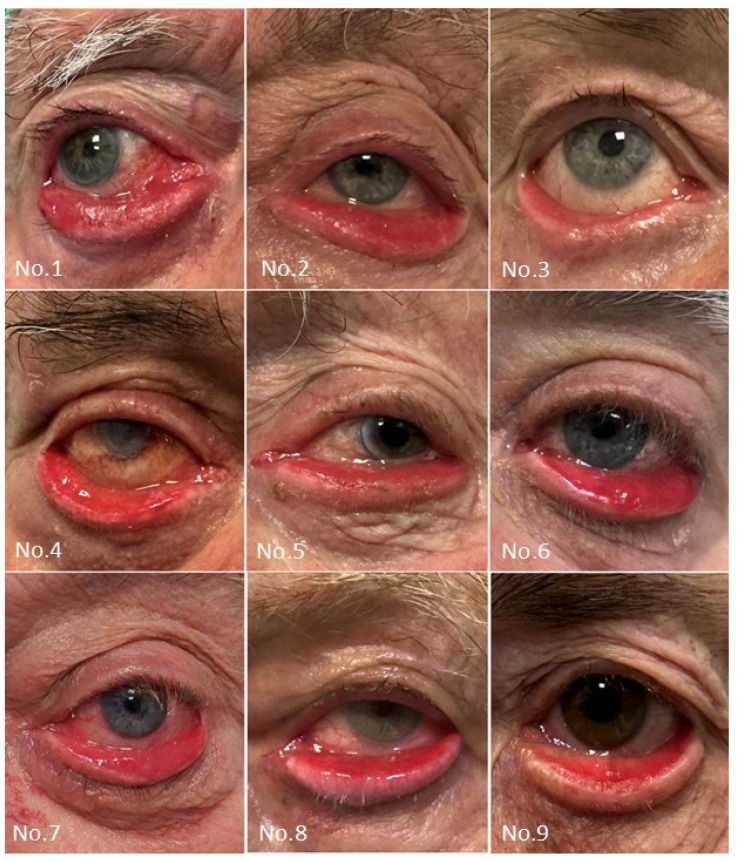
Preoperative clinical photographs of nine patients (No. 1–9) with long-standing severe involutional lower eyelid ectropion. All patients demonstrated complete lower eyelid eversion, marked exposure of the palpebral conjunctiva with pronounced hyperemia and varying degrees of keratinization, lateral canthal rounding, and punctal eversion.

A total of nine consecutive patients with unilateral severe involutional lower eyelid ectropion met the inclusion criteria during the study period. The cohort consisted of six men and three women, with a mean age of 81 years (range, 73–90 years). All patients presented with long-standing lower eyelid ectropion characterized by complete eyelid eversion, conjunctival hyperemia, variable degrees of keratinization, chronic epiphora, and ocular surface irritation (Figure 1). The duration of symptoms before surgery ranged from 3 to 22 months.

### 3.1. Primary Surgical Procedures

Horizontal eyelid shortening was performed in all patients using an LTS procedure. Additional medial conjunctival diamond excision was performed in four patients because of marked punctal eversion. Two patients required anterior lamella reconstruction using an ipsilateral upper eyelid transposition skin flap owing to skin deficiency. A temporary Frost suture was placed in two patients during the primary procedure.

Regarding vertical correction, three patients underwent complete lower eyelid retractor release during primary surgery because dense fibrosis, shortening, and persistent inferior traction of the retractors were identified intraoperatively despite correction of horizontal laxity. In the remaining six patients, the retractors were managed by reattachment or tightening according to conventional surgical principles. In some of these patients, no obvious fibrosis or shortening requiring release was recognized during the initial procedure, whereas in others, our surgical strategy had not yet evolved to include complete retractor release as a primary treatment option for advanced disease.

As experience accumulated during the study period, recurrent cases consistently demonstrated marked fibrosis and shortening of the lower eyelid retractors at revision surgery. These intraoperative findings led to a modification of our surgical approach, and complete retractor release subsequently became the preferred technique whenever advanced fibrosis and pathological inferior traction of the retractors were encountered.

Patient characteristics, surgical procedures, recurrences, and final outcomes are summarized in Table 1 and Table 2.

### 3.2. Recurrence and Revision Surgery (Table 2)

During a mean follow-up of 10.5 months (range 6–18 months), early recurrent ectropion developed in 5 of the 6 patients who underwent retractor reattachment or tightening as part of the primary procedure. No recurrence was observed in any patient who underwent complete retractor release during the initial operation.

**Figure 2:**
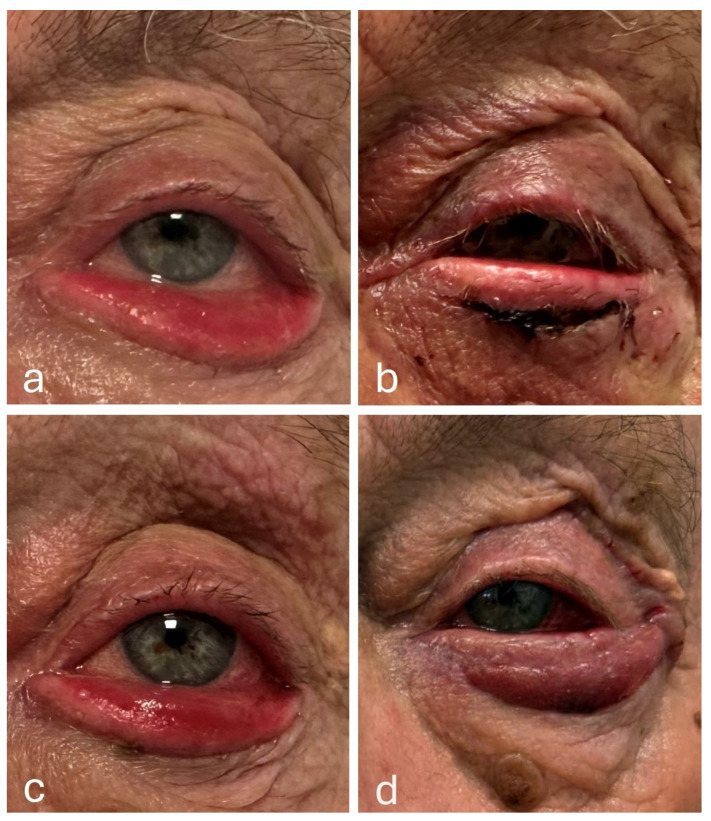
(**a**) Preoperative photograph of patient No. 2. (**b**) Early postoperative appearance 7 days after the primary lateral tarsal strip procedure combined with lower eyelid retractor reattachment. (**c**) Recurrent ectropion 5 months after the first revision procedure. The first revision was performed 2 months after the primary operation and consisted of wedge excision, medial conjunctival diamond excision, lower eyelid retractor tightening, and a Frost suture. (**d**) Postoperative appearance following the second revision surgery consisting of a lateral tarsal strip procedure, wedge excision, lower eyelid retractor release, transposition skin flap, and Frost suture, demonstrating a stable postoperative eyelid position without clinically relevant recurrent ectropion during the 7 months of follow-up.

Two patients (No. 2 and No. 7) with recurrent ectropion subsequently underwent two revision surgeries, the latest consisting of complete lower eyelid retractor release in combination with additional horizontal eyelid shortening procedures. In both patients, a stable postoperative eyelid position was maintained throughout the subsequent follow-up period without further recurrence (Figure 2). One additional patient (No. 8) underwent revision surgery at another institution. Two patients (No. 1 and No. 5) were considered candidates for further revision because of recurrent ectropion but were not reoperated because of significant medical comorbidities.

Overall, five patients underwent complete lower eyelid retractor release, either during the primary procedure (n = 3) or during revision surgery (n = 2). None of these patients developed recurrent ectropion during the available follow-up period.

In addition, all six patients who achieved stable eyelid position without recurrence (four after primary surgery and two after revision surgery) demonstrated satisfactory functional and aesthetic outcomes. Resolution or marked improvement of epiphora, ocular surface irritation, and conjunctival exposure was accompanied by restoration of stable eyelid–globe apposition and satisfactory cosmetic appearance.

Because of the retrospective design, limited sample size, heterogeneous surgical pathways, and absence of standardized functional outcome measures, no comparative statistical analysis between surgical techniques was performed.

## 4. Discussion

The present study describes our clinical experience with the surgical management of severe involutional lower eyelid ectropion presenting with complete eyelid eversion. Although limited by its retrospective design and small sample size, this case series suggests that complete lower eyelid retractor release may represent a useful adjunctive procedure in selected patients with advanced disease and intraoperative evidence of marked retractor fibrosis and shortening.

Involutional ectropion is generally regarded as a multifactorial disorder resulting from progressive age-related degeneration of the lower eyelid supporting structures. Horizontal eyelid laxity caused by attenuation of the medial and lateral canthal tendons, weakening of the orbicularis oculi muscle, and degeneration or disinsertion of the lower eyelid retractors all contribute to progressive eyelid malposition. Consequently, most established surgical techniques focus primarily on restoring horizontal eyelid stability, usually by means of a lateral tarsal strip procedure combined, when indicated, with retractor reattachment, tightening, or advancement [6,10,11,12,13,14,15,16,17,18].

The patients included in the present series, however, represented a distinctly different clinical population. All presented with long-standing disease characterized by fixed complete lower eyelid eversion, complete loss of eyelid–globe apposition, punctal eversion, and complete lateral canthal insufficiency manifested by rounding of the lateral canthus. In our opinion, these findings represent the end-stage of involutional ectropion rather than simply a more advanced degree of horizontal eyelid laxity.

**Figure 3:**
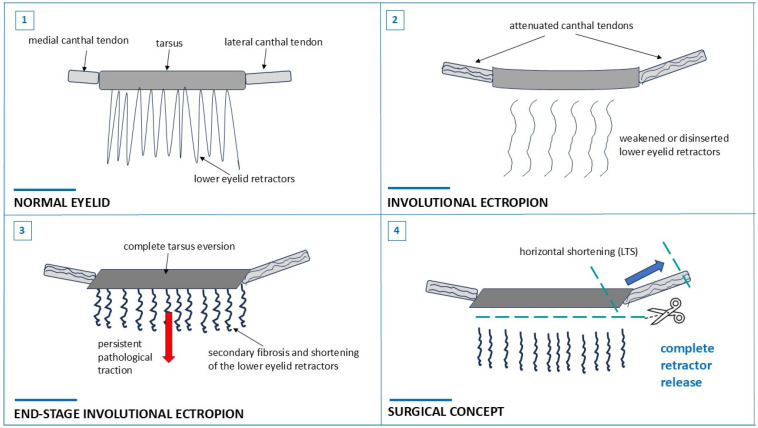
Schematic illustration of the hypothesized pathophysiological mechanism underlying end-stage involutional lower eyelid ectropion and the proposed rationale for complete lower eyelid retractor release. (**1**) Normal lower eyelid anatomy showing intact medial and lateral canthal tendons, the tarsal plate, and lower eyelid retractors. (**2**) Age-related involutional changes lead to attenuation of the canthal tendons and weakening or disinsertion of the lower eyelid retractors, resulting in progressive eyelid laxity and ectropion. (**3**) In long-standing disease, chronic eyelid eversion is hypothesized to promote secondary fibrosis and shortening of the lower eyelid retractors, producing persistent pathological inferior traction that maintains complete eyelid eversion despite correction of horizontal laxity. (**4**) Surgical concept: horizontal shortening using a lateral tarsal strip (LTS) procedure combined with complete release of the fibrotic lower eyelid retractors is hypothesized to reduce or eliminate pathological inferior traction, thereby facilitating restoration of normal eyelid–globe apposition.

We hypothesize that, in such advanced cases, the original involutional process becomes progressively complicated by secondary cicatricial remodeling. Chronic eyelid eversion exposes the palpebral conjunctiva to persistent desiccation, inflammation, and mechanical irritation, promoting progressive fibrosis of the posterior lamella. Histopathological studies have demonstrated degeneration of elastic fibers, collagen remodeling, and structural alterations within the lower eyelid retractors in involutional ectropion [4,19,21,22]. We speculate that prolonged disease duration may further promote fibrosis and shortening of the retractor complex, resulting in pathological inferior traction that actively maintains the everted eyelid position (Figure 3). Although supported by our intraoperative observations, this mechanism remains hypothetical, as no histopathological or biomechanical analyses were performed.

A particularly important observation in this series was the evolution of our surgical strategy during the study period. Initially, surgical management followed conventional principles, combining horizontal eyelid tightening with retractor reattachment or tightening when appropriate [15,16,17,18,20,23]. However, five of the six patients treated with this approach developed recurrent ectropion and subsequently required, or were considered candidates for, further surgical intervention. The only patient who remained free of recurrence was also the youngest in the series (73 years) and had the shortest duration of symptoms (3 months). Although no conclusions can be drawn from a single patient, it is plausible that the relatively short disease duration may have limited the development of secondary fibrotic changes within the posterior lamella. Consequently, restoration of normal retractor anatomy by reattachment or tightening may have been sufficient in this case.

In contrast, all remaining patients had a substantially longer history of ectropion, ranging from 8 to 22 months, and demonstrated marked intraoperative fibrosis, shortening, and reduced mobility of the lower eyelid retractors. During revision surgery, complete release of the fibrotic retractor complex eliminated the persistent inferior traction and allowed stable repositioning of the eyelid against the globe without recurrence during the available follow-up. These observations led to modification of our surgical approach. In the later patients included in this series, complete lower eyelid retractor release was therefore performed during the primary operation whenever advanced fibrosis and retractor shortening were identified intraoperatively.

Accordingly, the present study should not be interpreted as a comparison between retractor release and retractor reattachment or tightening. Rather, it describes the development of a modified surgical strategy based on progressively accumulated clinical experience in managing end-stage involutional ectropion. Within the limitations of this case series, no patient who underwent complete retractor release, either as a primary or revision procedure, developed recurrent ectropion during follow-up. Although encouraging, this observation cannot be interpreted as evidence of superiority over established techniques because treatment allocation was not standardized, the cohort was small, and several concomitant procedures were performed.

Interestingly, most published techniques addressing lower eyelid retractors aim to restore or reinforce their attachment to the inferior tarsal border by reattachment, tightening, or advancement [15,16,17,18,23]. Our observations suggest that this concept may not be applicable to all patients. In carefully selected individuals with long-standing end-stage involutional ectropion and marked secondary fibrosis, reinforcement of an already shortened and fibrotic retractor complex may fail to eliminate the pathological traction maintaining eyelid eversion. We therefore believe that the indication for complete lower eyelid retractor release should be determined by the stage of the disease rather than by the diagnosis of involutional ectropion alone. In early disease, preservation and reattachment of the retractors may remain appropriate, whereas in advanced long-standing ectropion with fixed eyelid eversion and intraoperative evidence of fibrosis, complete release may be a more suitable approach for eliminating pathological traction. Whether this concept applies only to the most advanced stages of involutional ectropion or to a broader patient population remains to be determined.

The present study has several limitations. It represents a small retrospective case series from a single institution without a predefined treatment algorithm. The indication for complete retractor release evolved during the study period as our understanding of the underlying pathology developed. Furthermore, standardized functional and patient-reported outcome measures, as well as objective grading of retractor fibrosis, were unavailable. Preoperative and postoperative photographs were obtained retrospectively from routine clinical documentation and were not acquired using a standardized photographic protocol. Finally, follow-up was relatively limited. Accordingly, our findings should be regarded as hypothesis-generating rather than evidence supporting a change in standard surgical practice.

## 5. Conclusions

In this preliminary retrospective case series, our observations are consistent with the hypothesis that end-stage involutional ectropion should not be regarded simply as advanced horizontal eyelid laxity. Instead, prolonged disease may result in secondary cicatricial remodeling of the lower eyelid retractors, creating a distinct pathological stage in which complete retractor release may be considered in selected patients rather than conventional reattachment. Recognition of this advanced disease stage may help surgeons identify patients who could benefit from complete lower eyelid retractor release. Prospective studies with larger patient cohorts are required to determine the role of this proposed surgical approach and to compare its outcomes with those of established techniques.

## Figures and Tables

**Table 1:** Baseline patient characteristics and primary surgical procedures.

Pt	Sex (M/F)	Age (yrs)	Duration (Months)	Horizontal Procedure	Vertical Procedure	Additional Procedures
1.	M	90	22	LTS	retractor tightening	medial conjunctival diamond excision transposition skin flap
2.	M	80	9	LTS	retractor reattachment	-
3.	F	75	13	LTS	retractor release	transposition skin flap
4.	M	79	8	LTS	retractor release	medial conjunctival diamond excision
5.	M	82	10	LTS	retractor tightening	Frost suture
6.	M	73	3	LTS	retractor tightening	-
7.	M	83	8	LTS	retractor reattachment	-
8.	F	87	15	LTS	retractor tightening	medial conjunctival diamond excision
9.	F	78	12	LTS	retractor release	medial conjunctival diamond excision

LTS = lateral tarsal strip.

**Table 2:** Recurrence, revision surgery, and final outcomes.

Pt	Recurrence	Time of Recurrence (Months)	Revision Surgery	Final Outcome	Follow-Up (Months)
1.	Yes	3	Not performed (medical comorbidities)	Persistent ectropion	15
2.	Yes	2	1. revision: wedge excision + medial conjunctival diamond excision + retractor tightening + Frost suture 2. revision: LTS + wedge excision + retractor release + transposition skin flap + Frost suture	Stable eyelid position after second revision	14 (7 after the second revision)
3.	No	-	-	Stable eyelid position	6
4.	No	-	-	Stable eyelid position	5
5.	Yes	2	Not performed (medical comorbidities)	Persistent ectropion	7
6.	No	-	-	Stable eyelid position	7
7.	Yes	3	1. revision: LTS + retractor tightening 2. revision: wedge excision + retractor release	Stable eyelid position after second revision	17 (9 after the second revision)
8.	Yes	3	Revision at another institution	Outcome unknown	18
9.	No	-	-	Stable eyelid position	6

LTS = lateral tarsal strip.

## Data Availability

The original contributions presented in this study are included in the article. Further inquiries can be directed to the corresponding author.

## Abbreviation

The following abbreviations are used in this manuscript:

LTS	Lateral Tarsal Strip
